# A novel E2F1-regulated lncRNA, LAPAS1, is required for S phase progression and cell proliferation

**DOI:** 10.18632/oncotarget.27962

**Published:** 2021-05-25

**Authors:** Esther Baruch, Tali Nizri-Megnaji, Oron Berkowitz, Doron Ginsberg

**Affiliations:** ^1^The Mina and Everard Goodman Faculty of Life Science, Bar-Ilan University, Ramat Gan, Israel; ^2^Azrieli Faculty of Medicine, Bar-Ilan University, Safed, Israel

**Keywords:** lncRNA, E2F, cell cycle, cell proliferation

## Abstract

The transcription factor E2F1 induces both proliferation and apoptosis and is a critical downstream target of the tumor suppressor RB.

Long non-coding RNAs (lncRNAs) are major regulators of many cellular processes, including cell cycle progression and cell proliferation. However, the mode of action as well as the transcriptional regulation of most lncRNAs are only beginning to be understood.

Here, we report that a novel human lncRNA, LAPAS1, is an E2F1- regulated lncRNA that affects S phase progression. Inhibition of LAPAS1 expression increases percentage of S phase cells, and its silencing in synchronized cells delays their progression through S phase. In agreement with its suggested role in cell cycle progression, prolonged inhibition of LAPAS1 attenuates proliferation of human cancer cells.

Our data demonstrate that LAPAS1 predominantly functions in trans to repress expression of Sphingolipid Transporter 2 (SPNS2). Importantly, knockdown of SPNS2 rescues the effect of LAPAS1 silencing on cell cycle and cell proliferation.

Notably, low levels of LAPAS1 are associated with increased survival of kidney cancer patients.

Summarily, we identify LAPAS1 as a novel E2F-regulated lncRNA that has a potential role in human cancer and regulates cell-cycle progression and cell proliferation, at least in part, via regulation of SPNS2.

## INTRODUCTION

The human genome expresses many thousands of long non-coding RNAs (lncRNAs), which are transcripts longer than 200 bases that lack a significant open reading frame [1]. They function as modulators of gene expression by controlling epigenetic modification, transcription, mRNA stability, translation as well as nuclear architecture [2–4]. Increasing evidence indicates that lncRNAs are key regulators of important biological processes including cell cycle progression [5], cell proliferation and apoptosis [6]. Specifically, some lncRNAs function in regulation of cell cycle progression via modulation of critical cell cycle players, such as the cyclins, CDKs, CDK inhibitors, pRB, and p53 [5, 7–9]. Aberrant expression of lncRNAs was detected in many tumors and lncRNAs were shown to contribute to the initiation and progression of numerous cancers [10–13] acting as tumor suppressors or oncogenes. Transcription factors that regulate mRNA transcription were shown to also regulate lncRNAs expression. These include the cancer-related transcription factors Myc [14–17], p53 [18–21], and E2F [22–30].

Here, we report the identification of a novel E2F1-regulated lncRNA, LAPAS1 (LncRNA whose silencing Attenuates Proliferation and S phase progression 1) that plays a role in S phase progression. We show that human lncRNA LAPAS1 levels are elevated upon activation of E2F1and knockdown of E2F1 reduces LAPAS1 levels. Moreover, expression of LAPAS1 is cell cycle regulated and peaks near G1/S transition and in early S phase. Inhibition of LAPAS1 expression delays progression of cells through S phase and inhibits proliferation of human cancer cells. Furthermore, LAPAS1 is a nuclear lncRNA and its silencing leads to a substantial rise in the levels of Sphingolipid Transporter 2 (SPNS2). Importantly, knockdown of SPNS2 rescues the effect of LAPAS1 silencing on cell proliferation and on cell cycle distribution at G1 and S. Notably, reduced levels of LAPAS1 are associated with increased survival of kidney cancer patients.

Thus, we identify LAPAS1 as a new E2F-regulated lncRNA that has a potential role in human cancer and regulates cell proliferation and cell-cycle progression, at least in part, via regulation of SPNS2.

## RESULTS

**Figure 1:**
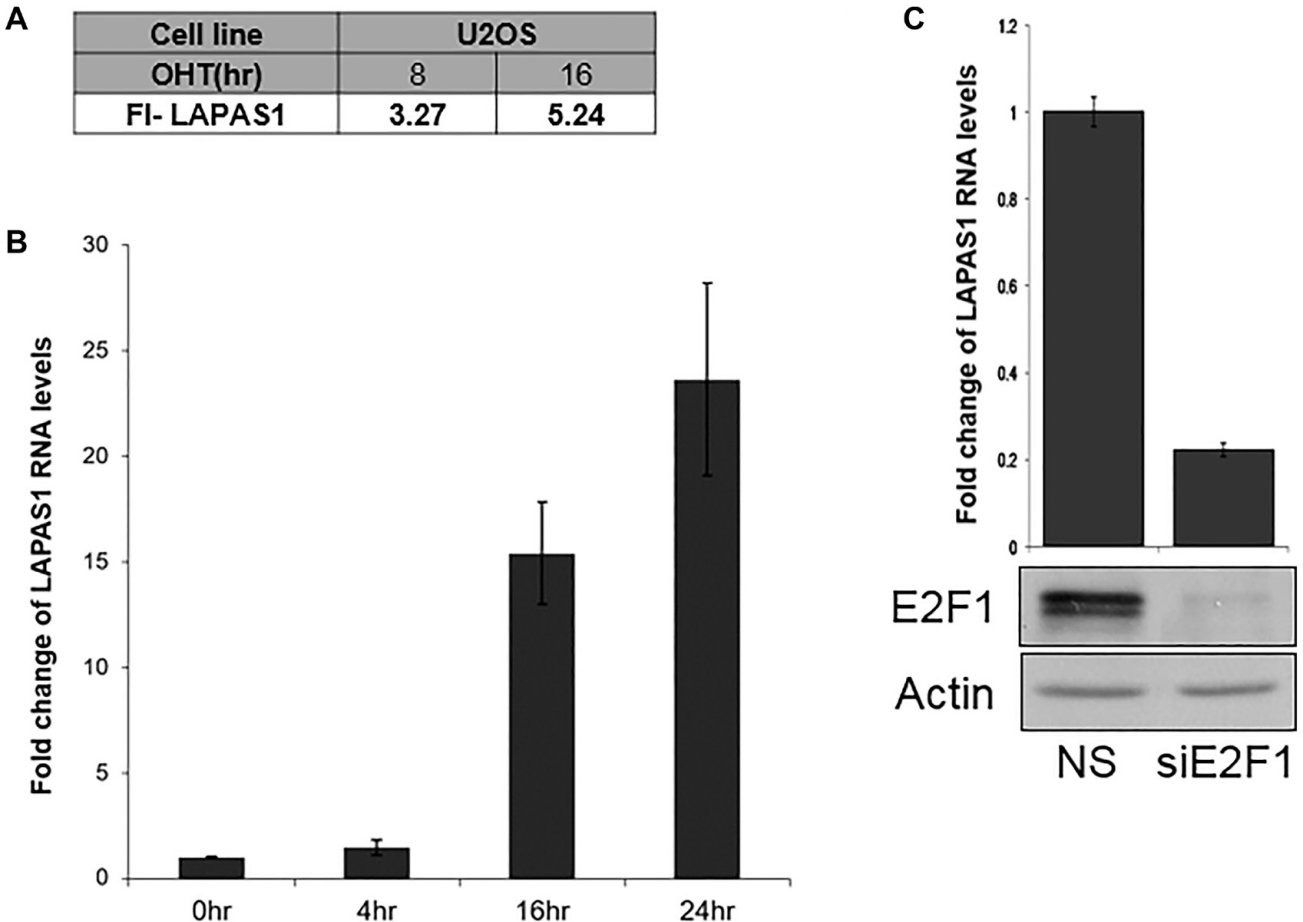
E2F1 regulates LAPAS1 RNA levels. (**A**) U2OS cells containing conditionally active E2F1 were induced to activate E2F1 by the addition of 4- hydroxytamoxifen (OHT) for the times indicated. RNA was extracted and RNA sequencing analysis was employed. FI-LAPAS1 represents fold increase in the number of reads for LAPAS1 after E2F1 induction. (**B**) U2OS cells containing conditionally active E2F1 were induced to activate E2F1 by the addition of OHT (times indicated). RNA was extracted, and LAPAS1 RNA levels were determined. (**C**) Upper panel-U2OS cells were transfected with either nonspecific siRNA (NS) or siRNAs directed against E2F1 (siE2F1). RNA was extracted and LAPAS1 RNA levels were determined by real-time RT-PCR and normalized to GAPDH levels. Lower panel-Proteins were extracted from cells and Western blot analysis was performed using antibodies directed against E2F1 and Actin.

**Figure 2:**
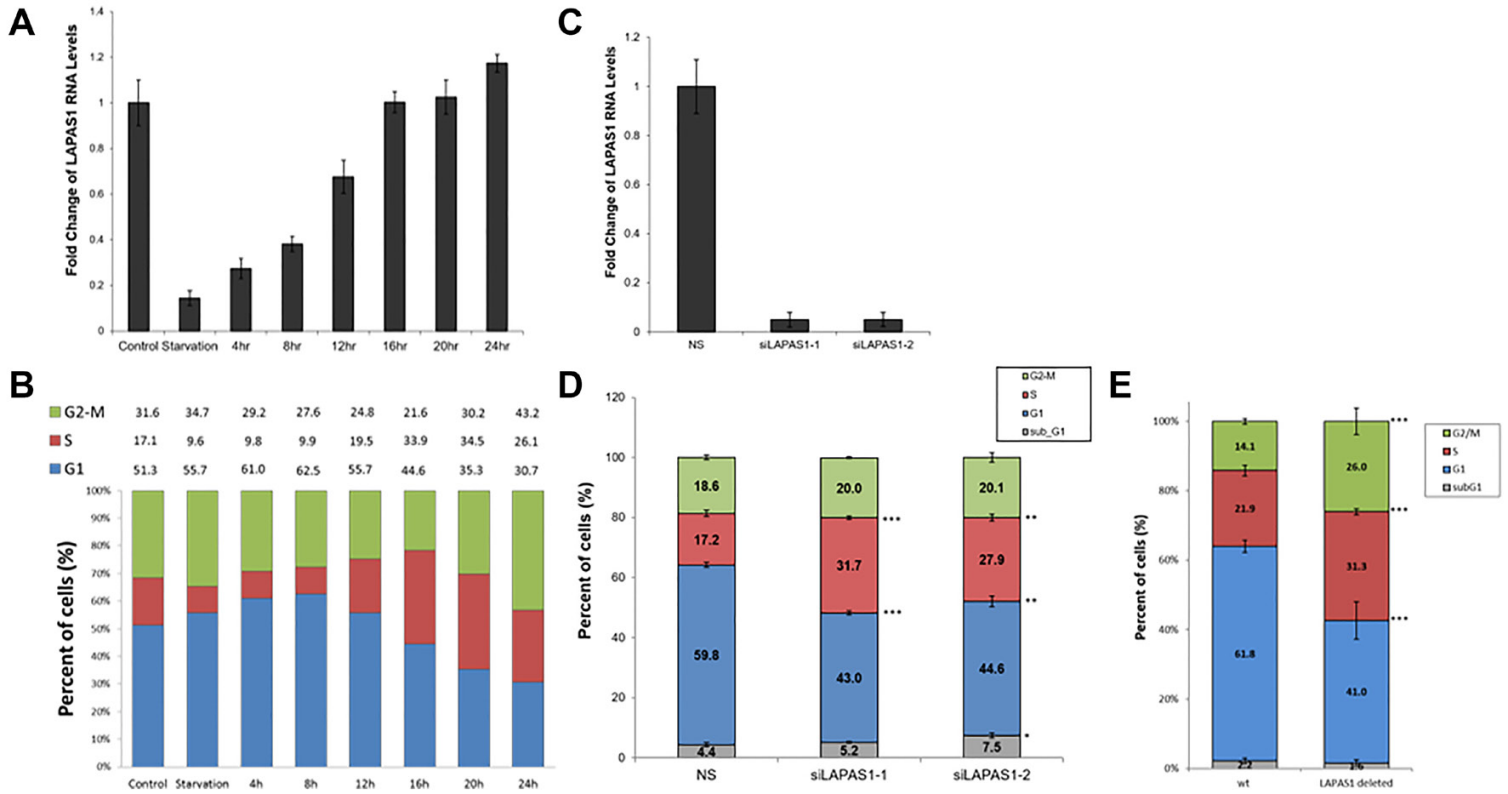
The RNA levels of LAPAS1 are cell cycle-regulated, and its inhibition results in cell cycle redistribution. U2OS cells were growth-arrested by serum deprivation (72 hours in medium without serum) and then allowed to resume growth by serum addition (to a final concentration of 15%) for the times indicated. (**A**) RNA was extracted and RNA levels of LAPAS1 were determined by real-time RT-PCR and normalized to GAPDH levels. (**B**) Cell cycle distribution was determined using FACS analysis. Percentage of cells in G1, S, and G2/M are indicated. U2OS cells were transfected with either a nonspecific siRNA (NS) or siRNAs directed against LAPAS1 (siLAPAS1-1 or siLAPAS1-2). (**C**) RNA was extracted and LAPAS1 RNA levels were determined by real-time RT-PCR and normalized to GAPDH levels. One representative experiment is shown out of 3 repeats. (**D**) Cells were analyzed by FACS using PI staining. An average of three independent FACS experiments is presented (^*^ *P* < 0.05, ^**^ *P* < 0.01, ^***^ *P* < 0.001; two-tailed Student’s *T*-test). (**E**) WT U2OS and LAPAS1-deleted cells were analyzed by FACS using PI staining. An average of three independent FACS experiments is presented (^***^ *P* < 0.001; two-tailed Student’s *T*-test).

LAPAS1 (also termed XLOC_000190) is a 35,375 bases long intergenic lncRNA, which is located on chromosome 1 (chr1:51,443,233-51,479,174). It is transcribed from the plus strand and consists of three exons and its mature RNA is 4,338 bases long. Of note, this lncRNA has additional isoforms, for example NONHSAG001438.3 that is annotated in the NONCODE database. However, detailed PCR analysis with a number of primers demonstrated that the above-mentioned transcript is the only one expressed in the cells used in this study (data not shown) and it was therefore the one further studied. Initially we identified LAPAS1 as an E2F1-regulated lncRNA in an RNA seq-based screen. This screen employed human osteosarcoma cells that express an inducible E2F1. This analysis showed that activation of inducible E2F1 led to a 3.2 and 5.2 fold increase in the RNA levels of LAPAS1after 8 and 16 hours, respectively (Figure 1A). Information from the UCSC browser indicates that the putative promoter of LAPAS1 binds E2F1 and we detect 5 putative E2F binding sites in this region. Validation of the RNA-seq data by real-time PCR analysis showed that induction of E2F1 increased expression of LAPAS1 by up to 24 fold (Figure 1B). Moreover, knockdown of E2F1 resulted in a substantial decrease in RNA levels of LAPAS1 (Figure 1C) indicating that endogenous E2F1 regulates LAPAS1 levels. E2F1 is a crucial controller of cell cycle progression and numerous E2F-regulated genes are expressed in a cell cycle-regulated manner. Therefore, we tested whether LAPAS1 is differentially expressed during cell cycle progression. Specifically, we tested LAPAS1 levels in cells that were growth-arrested at G1 phase by serum starvation and then reentered the cell cycle upon the addition of serum. Upon serum starvation, LAPAS1 levels dropped by 7 fold, and this was followed by gradual elevation as cells reentered the cell cycle (Figure 2A). Specifically, we detected a 2-2.5 fold increase in LAPAS1 levels 4–8 hours after serum addition and this increase occurred prior to G1/S transition (Figure 2B). As the percentage of cells in S phase increased 12 and16 hours after serum addition, LAPAS1 levels increased 4 and 7 fold, respectively.

**Figure 3:**
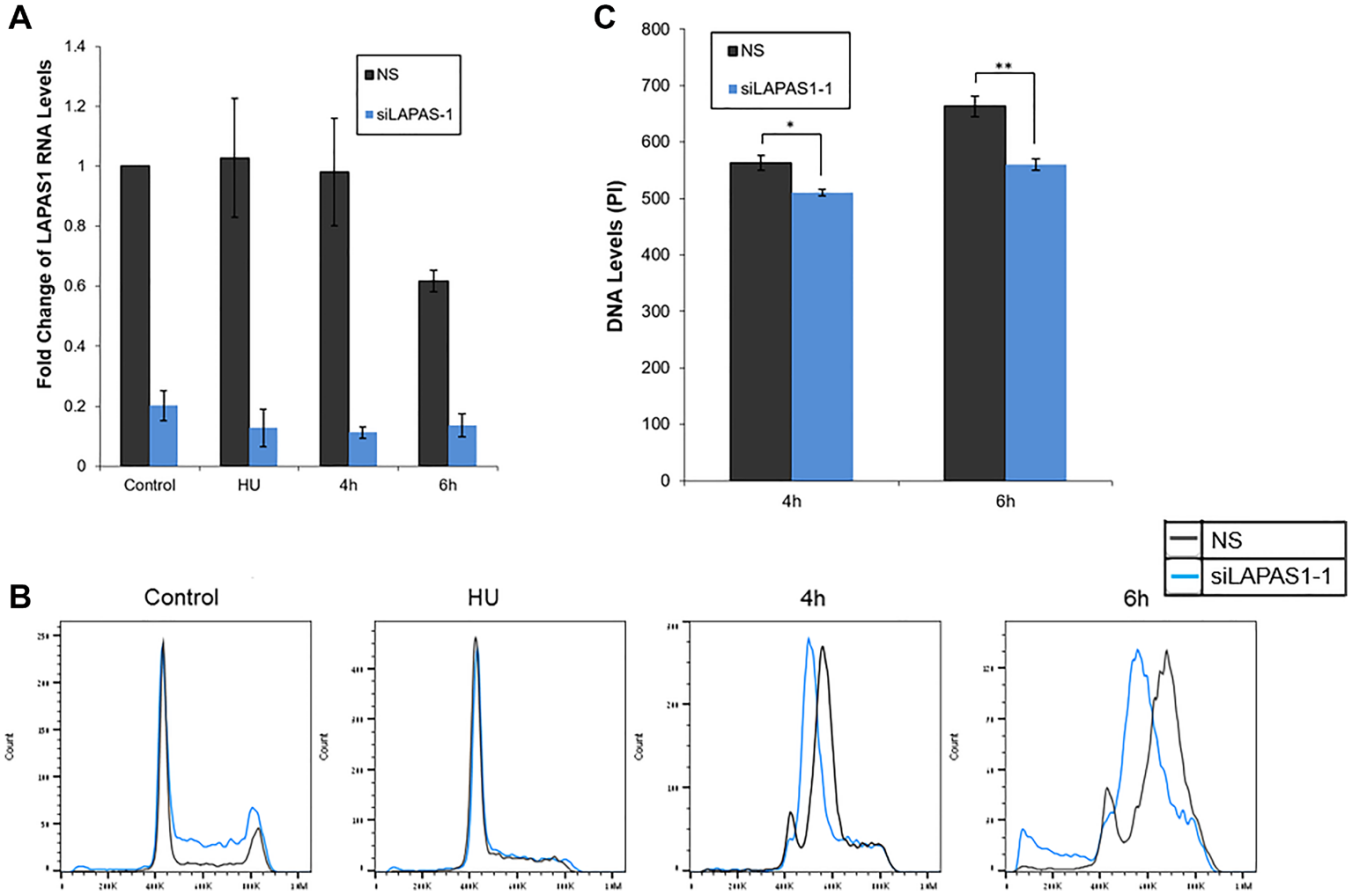
Silencing of LAPAS1 in G1-synchronized U2OS cells results in slower progression through S phase. U2OS cells were transfected with either a nonspecific siRNA (NS) or siRNA directed against LAPAS1 (siLAPAS1-1). Next, cells were incubated with hydroxyurea (4 mM) for 20 hours. 48 hours post-transfection, cells were harvested or allowed to resume growth by incubation in fresh media for times indicated. (**A**) RNA was extracted and LAPAS1 RNA levels were determined. An average of three independent experiments is presented. (**B**) Cells were analyzed by FACS using PI staining. One representative experiment is shown. (**C**) The PI levels at the peaks of S phase are presented. An average of three independent FACS experiments is presented (^*^ *P* < 0.05, ^**^ *P* < 0.01 two-tailed Student’s *T*-test).

Numerous E2F target genes encode proteins that regulate cell cycle progression [31]. Also, some E2F-regulated ncRNAs regulate cell cycle progression [22, 23, 27, 28, 32–34]. Therefore, we examined whether LAPAS1 plays a role in cell cycle progression. Indeed, decreasing endogenous LAPAS1 RNA levels using two distinct siRNAs led to a redistribution of cells in the cell cycle (Figure 2D). Specifically, knockdown of LAPAS1 (Figure 2C) led to a substantial reduction in the percentage of cells in the G1 phase and a simultaneous increase in the percentage of cells in S phase (Figure 2D). This change in cell cycle distribution was reproducible and statistically significant (Figure 2D), suggesting that LAPAS1 silencing may enhance S phase entry and/or attenuate the progression of cells through the S phase. Similarly, deletion of one allele of LAPAS1 in U2OS cells (Verified by sequencing Supplementary Figure 1) also led to a substantial reduction in the percentage of cells in the G1 phase and a simultaneous increase in the percentage of cells in S phase (Figure 2E). In order to further establish the role of LAPAS1 in S phase, U2OS cells were treated with hydroxyurea (HU) that blocks cells at the G1/S boundary. The cells were then released from the hydroxyurea-induced arrest, and their progression in the cell cycle was analyzed. Upon release from the hydroxyurea block, LAPAS1’s silenced cells progressed more slowly through S phase than cells transfected with nonspecific siRNA (Figure 3A and 3B). Also, FACS analysis of the cells released from HU-block demonstrated that the DNA content of LAPAS1-silenced cells is significantly lower than that of cells transfected with nonspecific siRNA (Figure 3C). Similar experiments using another siRNA that targets LAPAS1 also demonstrated that knockdown of LAPAS1 inhibits the progression of cells through S phase (Supplementary Figure 2). Taken together, our data indicate that the lncRNA LAPAS1 most probably functions in regulating cell cycle progression, and, specifically, S phase progression.

**Figure 4:**
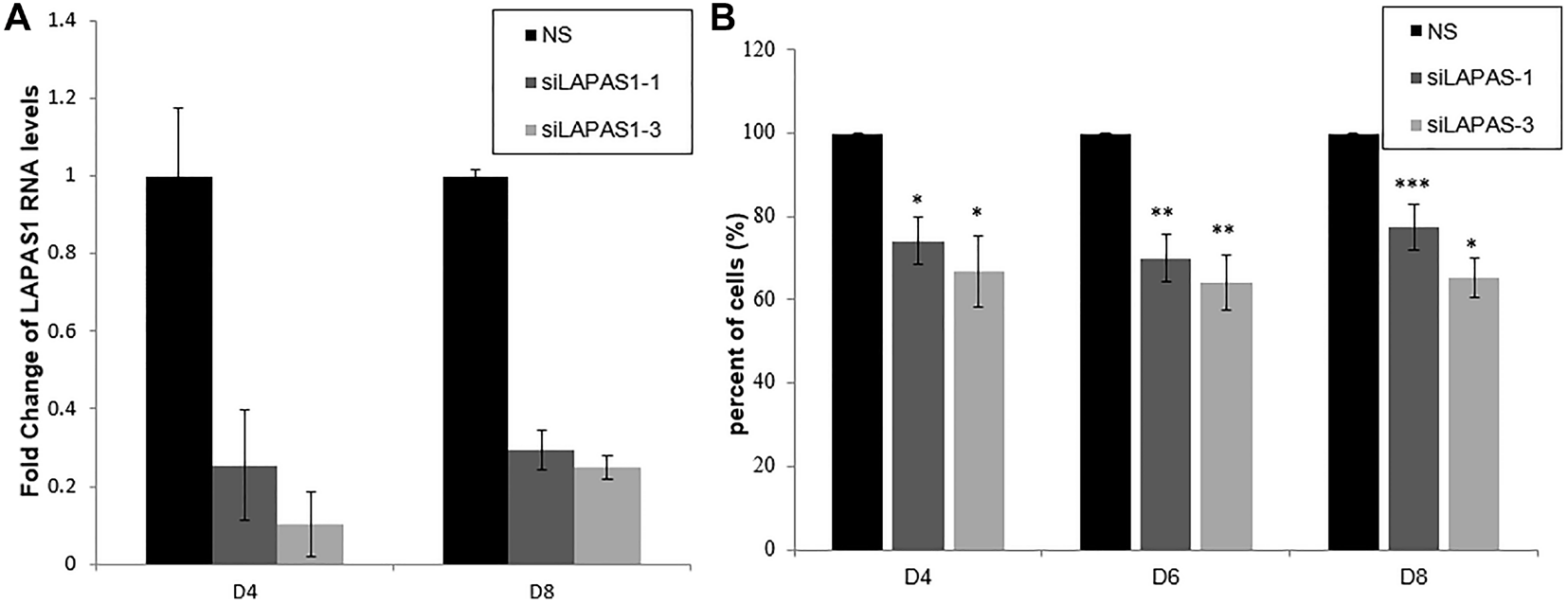
Prolonged silencing of LAPAS1 inhibits cell proliferation. U2OS cells were transfected with either a nonspecific siRNA (NS) or siRNAs directed against LAPAS1 (siLAPAS1-1 or siLAPAS1-3). Cells were harvested at indicated days post-platting. (**A**) RNA was extracted and LAPAS1 RNA levels were determined. (**B**) 24 h post-transfection, an equal number of cells was seeded. Cells were trypsinized and counted at indicated days post-plating. The number of cells of NS cells is depicted as 100%. An average of three independent experiments is presented (^*^ *P* < 0.05, ^**^ *P* < 0.01, ^***^ *P* < 0.001; two-tailed Student’s *T*-test).

**Figure 5:**
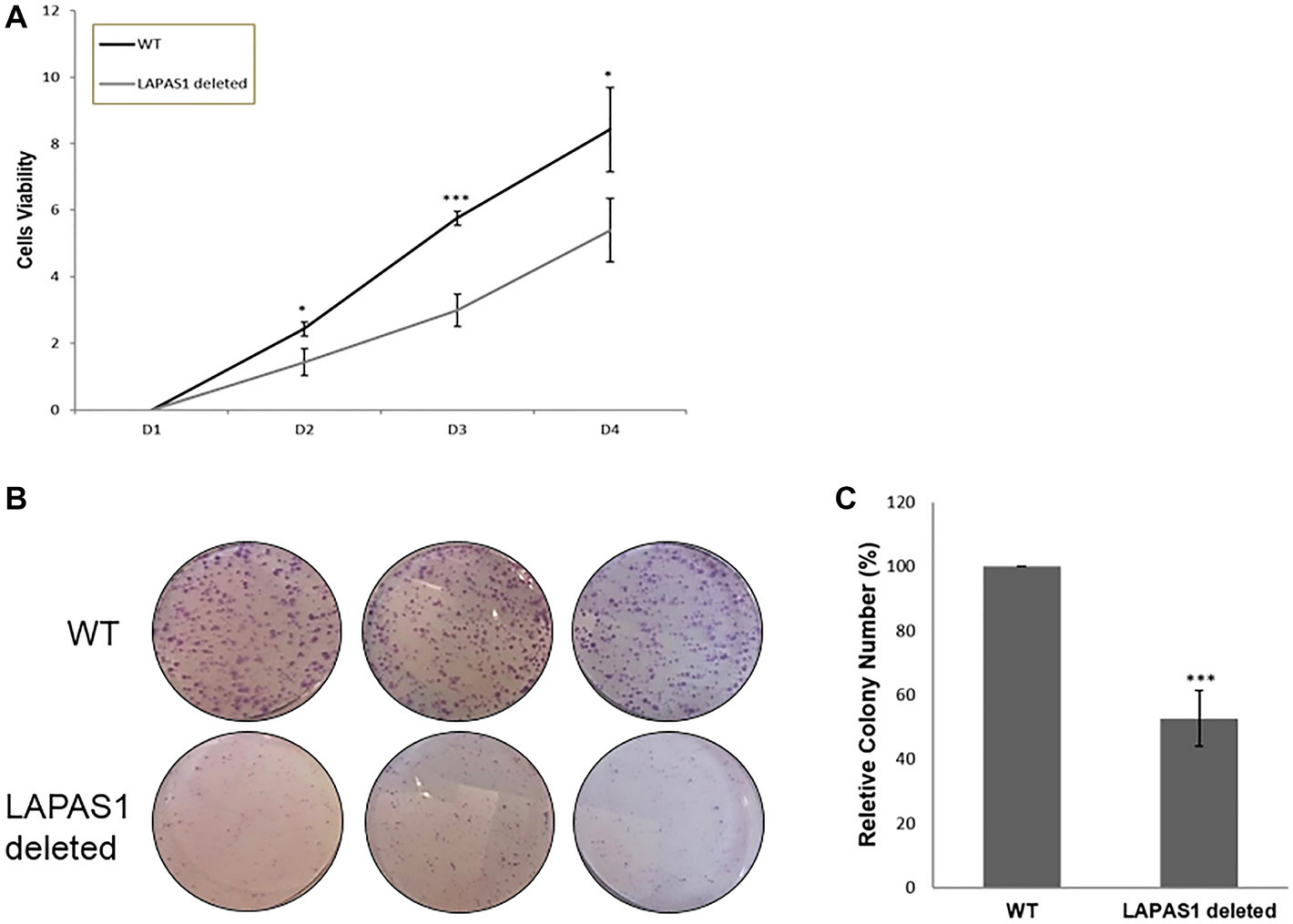
LAPAS1 deletion attenuates cell proliferation. (**A**) An equal number of WT and LAPAS1-deleted U2OS cells were seeded and cultured for indicated times (in days). Cell proliferation was assessed by MTT assay. An average of three independent experiments is presented (^*^ *P* < 0.05, ^***^ *P* < 0.001; two-tailed Student’s *T*-test). (**B**) Colony formation assay of WT (upper three plates) and LAPAS1-deleted (lower three plates) U2OS cells. (**C**) The average relative number of colonies of three independent experiments is presented. The number of colonies of WT cells is depicted as 100% (^***^ *P* < 0.001; two-tailed Student’s *T*-test).

Next, we tested the effect of prolonged silencing of LAPAS1 or its heterozygous deletion on cell proliferation and viability. As seen in Figure 4A, substantial reduction of LAPAS1 levels was maintained for at least 8 days after transient transfection of siRNA. Cell counting demonstrated that this prolonged silencing of LAPAS1 inhibited cell growth to 65–75% of wt cells over 8 days (Figure 4B). Similarly, deletion of one allele of LAPAS1 also resulted in reduced cell proliferation as determined by an MTT assay. Specifically, this deletion of one allele of LAPAS1 led to a 35% decrease in the number of viable cells after four days of culturing (Figure 5A). In addition, this deletion of LAPAS1 resulted in a 2-fold decrease in the number of colonies, as determined by a colony assay (Figure 5B and 5C). These data strongly suggest that LAPAS1 plays a role in cell proliferation and they are consistent with the effect of LAPAS1 silencing or heterozygous deletion on cell cycle distribution, as shown above (Figures 2 and 3).

**Figure 6:**
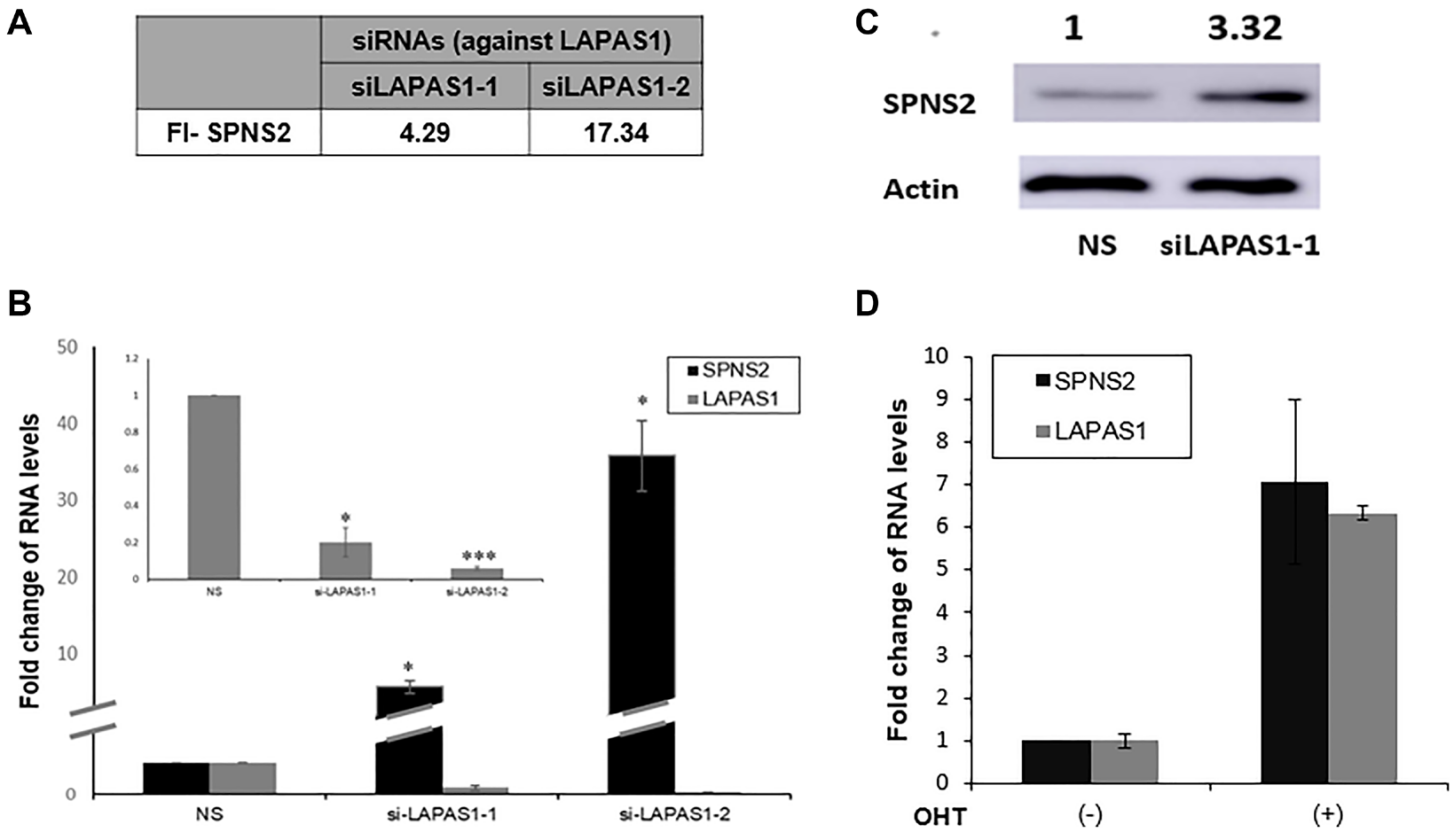
SPNS2 levels increase following LAPAS1 silencing or E2F1 activation. (**A**) U2OS cells were transfected with either a nonspecific siRNA (NS) or siRNAs directed against LAPAS1 (siLAPAS1-1 or siLAPAS1-2). RNA was extracted and RNA sequencing analysis was employed. FI-SPNS2 represents a fold increase in the number of reads for SPNS2 after LAPAS1 silencing. (**B**) RNA was extracted from cells treated as described in (A). Next, RNA levels of Spns2 and LAPAS1 (inner bar graph) were determined. An average of three independent experiments is presented (^*^ *p* < 0.05, ^***^ *p* < 0.005 two-tailed Student’s *t*-test). (**C**) Proteins were extracted from cells treated as described in A and Western blot analysis was performed using antibodies directed against SPNS2 and ACTIN. (**D**) U2OS cells expressing ER-E2F1 were left untreated or incubated with OHT (100 nM) for 8 hr. RNA was extracted and SPNS2 and LAPAS1 RNA were determined.

In an attempt to elucidate the mechanism underlying the effects of LAPAS1 on cell proliferation and cell cycle progression, we first analyzed its subcellular localization. Fractionation experiments demonstrated that LAPAS1 is mainly nuclear (Supplementary Figure 3). Many nuclear lncRNAs affect gene expression and, therefore, we employed RNA-seq. analysis to search for genes whose expression was altered upon silencing of LAPAS1 (Supplementary Table 1). This analysis identified the gene SPNS2 (Sphingolipid Transporter 2), which was significantly upregulated upon silencing of LAPAS1 with two different siRNAs (Figure 6A). These RNA- seq data were validated by real-time PCR (Figure 6B). Furthermore, the protein levels of SPNS2 were also elevated upon knockdown of LAPAS1 (Figure 6C). Of note, mRNA levels of SPNS2 increased upon activation of inducible E2F1 (Figure 6D) demonstrating that both SPNS2 and LAPAS1, which negatively regulates SPNS2, are E2F targets. Thus, E2F, LAPAS1 and SPNS2 constitute an incoherent feed-forward loop, which is a common motif in transcriptional networks [35].

**Figure 7:**
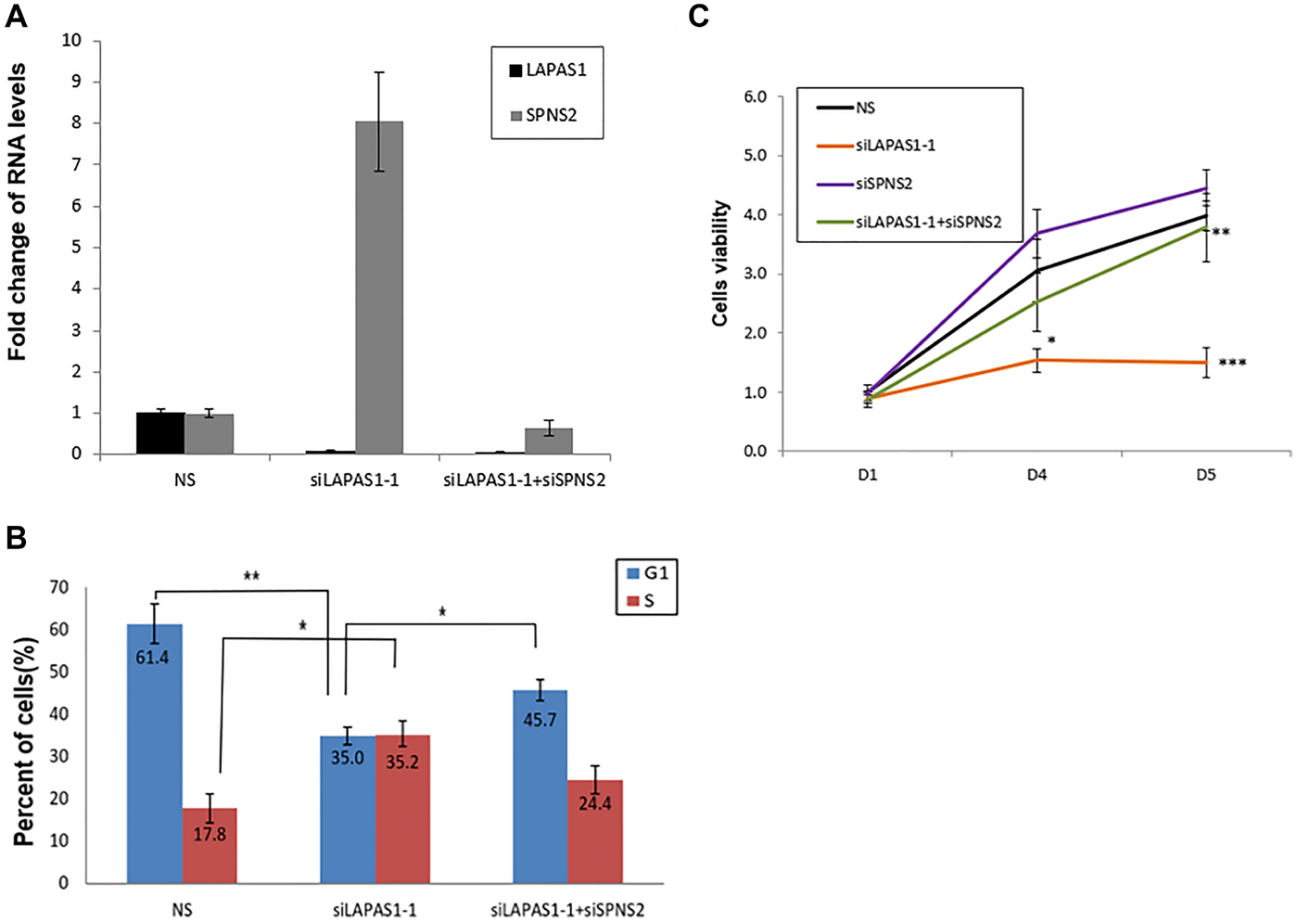
Silencing of SPNS2 rescues the effect of LAPAS1 silencing on cell cycle progression and cell proliferation. U2OS cells were transfected with either a nonspecific siRNA (NS), siRNA directed against LAPAS1 (siLAPAS1-1) or siRNA directed against LAPAS1 and SPNS2 (siLAPAS1-1 + siSPNS2). (**A**) RNA was extracted and LAPAS1 and SPNS2 RNA levels were determined. One representative experiment is shown out of 3 repeats. (**B**) Cells were analyzed by FACS using PI staining. An average of three independent FACS experiments is presented (^*^ *p* < 0.05,^**^ *p* < 0.01 two-tailed Student’s *t*-test). (**C**) An equal number of U2OS cells were seeded and transfected with either nonspecific siRNA (NS), siRNA targeted against LAPAS1 (siLAPAS1-1), siRNA against SPNS2, or both. Cells were grown for the indicated times (in days). Cell proliferation was measured by MTT assay. An average of four independent experiments is presented (^*^ *p* < 0.05, ^**^ *p* < 0.01, ^***^ *p* < 0.005 two-tailed Student’s *t*-test).

To investigate the possibility that upregulation of SPNS2 mediates the effects of LAPAS1 knockdown on cell cycle and cell proliferation, we co-silenced LAPAS1 and SPNS2 (Figure 7A). As observed previously, silencing of LAPAS1 resulted in fewer cells in G1 phase and more cells in S phase (Figure 7B). Noticeably, co-silencing of SPNS2 partially rescued this effect, that is, the percentage of cells in S phase of cell cycle decreased and the percentage of cells in G1 phase increased compared to LAPAS1 knockdown alone (Figure 7B and Supplementary Figure 4). Similar results were obtained when LAPAS1 was silenced using a different siRNA (Supplementary Figure 5). To test whether SPNS2 also mediates the effect of LAPAS1 silencing on viability as well as cell proliferation we used an MTT assay. This analysis demonstrated that prolonged silencing of LAPAS1 significantly reduces the increase in the number of viable U2OS cells over time (Figure 7C). Importantly, co-silencing of SPNS2 (Supplementary Figure 6) partially rescued this inhibition (Figure 7C), indicating that indeed SPNS2 mediates the effect of LAPAS1 knockdown on viability and cell proliferation.

**Figure 8:**
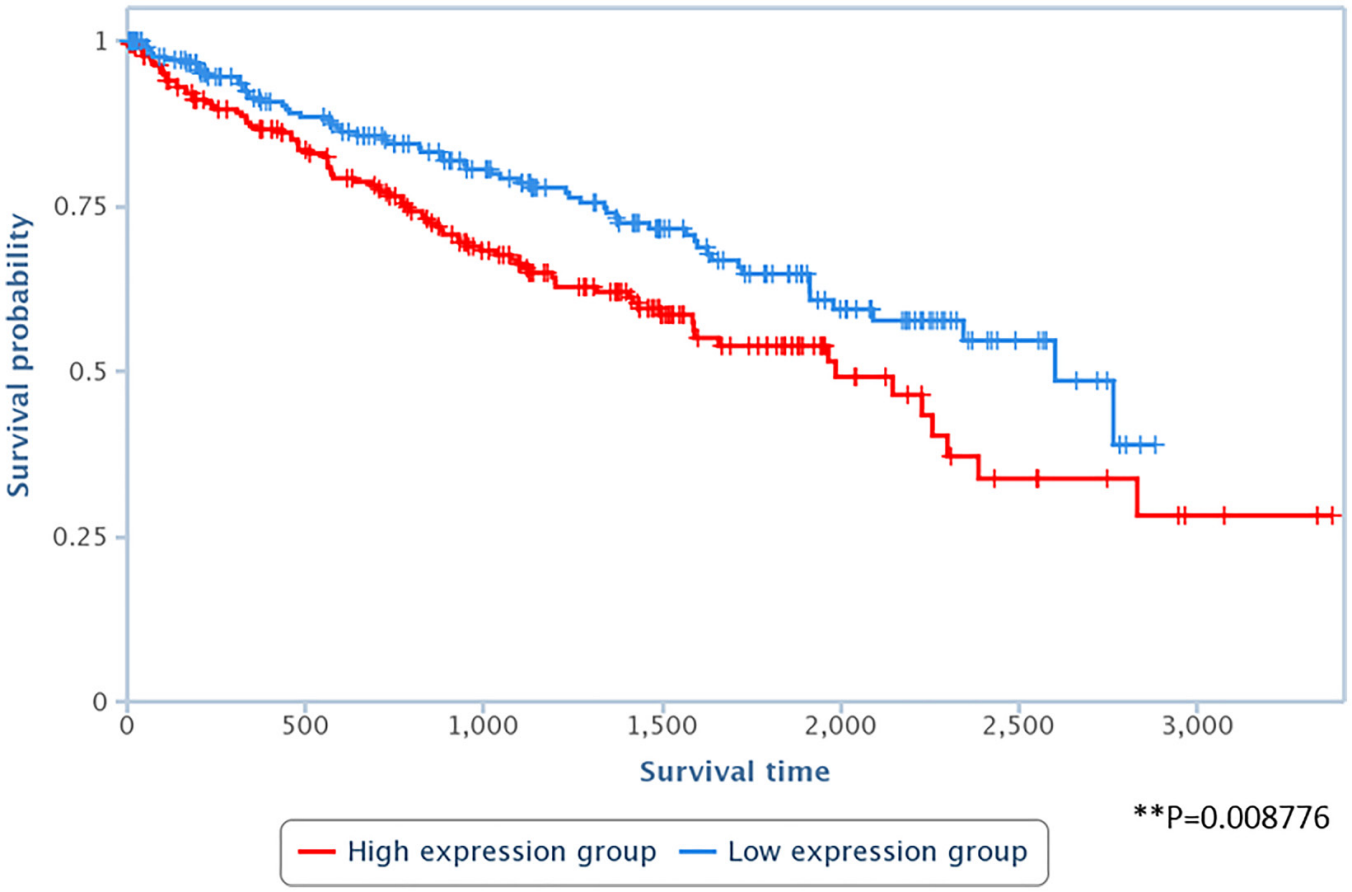
High levels of LAPAS1 are associated with poor prognosis in kidney cancer patients. RNA-seq data derived from 448 kidney renal clear cell carcinoma (KIRC) cancer samples were divided into two subgroups, according to the RNA levels of LAPAS1 (high vs. low). LAPAS1 expression was based on reads within chr1:51,443,233–51,479,174. *P* < 0.01.

Lastly, to assess the relevance of LAPAS1 to human tumors, we tested whether its levels correlate with patient survival in a group of 448 kidney renal clear cell carcinoma (KIRC) cancer samples. Interestingly, upon stratification of this group of patients to two subgroups: the first with high levels of LAPAS1 and the second with low levels of LAPAS1, it became clear that the subgroup with low levels of LAPAS1 displayed increased survival (Figure 8). These data are in agreement with our findings that reduction of LAPAS1 levels inhibits cell growth (Figures 4 and 5).

## DISCUSSION

E2Fs are transcription factors that regulate the timely expression of protein-coding genes that function in cell cycle progression [31]. E2F1 is best known as a positive regulator of the G1/S transition, however, expression of some critical regulators of other cell cycle stages is also regulated by E2F1 [36–38]. Although previous studies on E2F’s transcriptional network have mainly focused on protein-coding genes, it has been increasingly recognized that E2F is also capable of regulating noncoding RNAs, including both microRNAs [39–42] and long noncoding RNAs. Thus far, expression of at least ten lncRNAs was shown to be regulated by E2F [22–29, 32–34]. These include lncRNAs that were reported to affect cell cycle progression, such as ANRIL, that repressed the expression of the CDK inhibitors p16Ink4a and p15Ink4b [28, 33, 34, 43]; MA-linc1, which regulates M/G1 transition and enhances Paclitaxel-induced apoptosis [27]; LINC00668, which, like ANRIL, enhances cell proliferation via silencing of CDK inhibitors [32]; GASL1, which restrains cell cycle progression as well as cell proliferation [23]; and LINC00673, which regulates cellular senescence in lung cancer and affects G1/S transition [22].

The current work reveals LAPAS1 as a new E2F-inducible lncRNA. Like numerous E2F targets, expression of LAPAS1 is cell cycle regulated and peaks near G1/S transition and in early S phase. Silencing or deletion of one allele of LAPAS1 delays progression of cells through S phase and inhibits proliferation of human cancer cells, indicating that it functions as a regulator of cell cycle progression and proliferation. LAPAS1 silencing leads to a significant increase in the levels of Sphingolipid Transporter 2 (SPNS2) mRNA and protein. Importantly, knockdown of SPNS2 partially rescues the effect of LAPAS1 silencing on S phase progression and cell proliferation, strongly suggesting that LAPAS1 affects cell cycle progression and proliferation, at least in part, by regulating SPNS2 expression.

Sphingolipid Transporter 2 (SPNS2) is an S1P transporter that is best known for its roles in the survival and migration of cells, including cancer cells [43, 44]. Our data indicate that it may also function as a regulator of cell proliferation, and are consistent with previous studies showing that modulation of its levels affects cell proliferation [45] and its loss interferes with cell cycle exit [46]. Also, SPNS2 was previously shown to be associated with cell proliferation by gene ontology analyses [47].

Our study shows that silencing the lncRNA LAPAS1 upregulates SPNS2 levels. Recently, another lncRNA, lncRNA-5657, was shown to bind the promoter of SPNS2 and enhance its expression [48]. Taken together with our data, this indicates that regulation of SPNS2 is complex and depends on the relative expression of two distinct lncRNAs. The chromosomal location of SPNS2 gene on chromosome 17 (chr17:4402176-4442330) suggests that LAPAS1 affects it in trans. Sequence analysis of LAPAS1 and the Spns2 promoter and the Spns2 mRNA did not detect any significant sequence identity or complementarity. Thus, the molecular mechanism by which LAPAS1 affects the expression of SPNS2 is currently unknown and awaits further studies. Of note, an effect of LAPAS1 in cis on its neighboring gene, which encodes the CDK inhibitor p18InkD, has been ruled out, as silencing of LAPAS1 did not affect p18^IknD^ mRNA levels (Supplementary Figure 7).

Interestingly, we show here that E2F upregulates the expression of both the lncRNA LAPAS1 and the protein-coding gene SPNS2. In addition, our data strongly suggest that LAPAS1 negatively regulates SPNS2 expression. Thus, E2F, LAPAS1 and SPNS2 constitute an incoherent type 1 feed-forward loop (FFL), whereby a transcription activator activates directly a gene as well as its repressor [49]. Similar FFLs have been previously demonstarted in the E2F pathway. For example, E2F1 regulates the expression of miR499a/b, which negatively regulate Cdk6 and Cdc25a that are themselves E2F targets [42]. Also, E2F1 regulates the levels of miR-15 and Mir-16, which in turn negatively regulate cyclin E, another E2F target [39].

E2F regulates the expression of another key enzyme in the S1P pathway, Sphk1 and also this regulation involves a FFL with an lncRNA. E2F1 upregulates the expression of the lncRNA Khps1, which then binds the Sphk1 promoter and enhances activation of the Sphk1 gene by E2F1 [25]. Thus, this is a coherent FFL, unlike the regulation that we report here, which is an incoherent FFL. Nevertheless, it is of great interest that E2F regulates two key proteins in the S1P pathway and in both cases there is involvement of an E2F-regulated lncRNA.

It has been increasingly recognized that lncRNAs play an important role in the regulation of cell cycle progression in general and E2F activity in particular. Therefore, identification and functional analysis of a new E2F-regulated lncRNA, LAPAS1, is of great importance to the understanding of the control of cell cycle progression and cell proliferation. LAPAS1 is expressed in a number of tissues and cancer samples. In agreement with its role in cell cycle progression and cell growth, the study of kidney cancer patients indicates that LAPAS1 is relevant in human cancer as low levels of LAPAS1 correlate with improved survival of these patients.

In summary, this study reports the identification of a novel lncRNA that affects cell cycle progression and cell proliferation and may affect cancer progression. Its initial characterization shows that it is transcriptionally regulated by E2F and it exerts its activity, at least in part, by regulating SPNS2.

## MATERIALS AND METHODS

### Cell culture

U2OS cells were grown as previously described [23]. Where indicated, hydroxyurea was added at 4 mM for 20 hr.

### Plasmids

The plasmid pBabe-neo-HA-ER-E2F1was used. For CRISPR/Cas9-mediated deletion pSpCas9(BB)-2A-GFP was used.

### Quantitative PCR (Real-Time RT- PCR)

Real-Time RT- PCR was performed as previously described [23] with the following primer pairs:

GAPDH: 5′-CATGTTCCAATATGATTCCACC and 5′-GATGGGATTTCCATTGATGAC.

LAPAS1: 5′-TGAACACAAAAACAGGTCCAA and 5′-TCTTCTGCATTCAAAATTCCAA.

SPNS2: 5′-CTGCTTTACGGGATTTCTGG and 5′-CACGAAGATCAGGCAGATGA

CDKN2C: 5′-CCGATTTGAAAGACCGAACT and 5′-GGGCAGGTTCCCTTCATTAT.

MALAT1: 5′-TGGGGGAGTTTCGTACTGAG and 5′- TCTCCAGGACTTGGCAGTCT.

7sl2: 5′- CAAAACTCCCGTGCTGATCA and 5′- GGCTGGAGTGCAGTGGCTAT.

RNA levels of LAPAS1, SPNS2 and CDKN2C were normalized to GAPDH levels. Results are presented as mean and SD for duplicate runs.

### Transfection

Transfection of plasmids was performed as previously described [23]. Transfection of siRNAs was performed using Interfferin transfection reagent (PolyPlus-transfection), according to the manufacturer’s instructions.

The siRNAs indicated below and a control sequence (siRNA universal negative control #1) were synthesized by Sigma-Aldrich. Cells were harvested 48 hours post siRNAs transfection.

siRNA Sequence:

SiE2F1: CAGAGCAGAUGGUUAUGGU

SiLAPAS1_1: GAUGCCAGGUAGAUUAGGUUAUUAA

SiLAPAS1_2: ACCACACGTGCATGCTACCACATCT

SiLAPAS1_3: GGACAGAUUCAAAUCGCCUAACAUA

Si_SPNS2-GCGACCGCTTCAACAGGAAGGTGAT

### Fluorescence-activated cell sorting (FACS) analysis

FACS analysis was performed as previously described [23].

### Cell proliferation and viability (MTT) assay

2,000 U2OS cells were seeded into 96-well plates as tetraplicates. On the following day, siRNAs were transfected as described above and the cells were grown for the indicated number of days. Then, cells were stained with MTT (3-[4,5-dimethylthiozol-2-yl]-2,5diphenyltetrazoliumbromide) by incubating for 30 min at 37°C in the dark. OD was measured at absorbance of 570 nm using a TECAN spectrophotometer. Per each day and each clone, tetraplicates of OD were obtained and averaged.

### Western blotting

Cell pellet preparation and western Blot analysis were performed as previously described [27]. The membrane was incubated with one of the following primary antibodies: anti-E2F1 (sc-251, Santa Cruz Biotechnology); anti-Actin (sc-1616r; Santa Cruz Biotechnology); anti-SPNS2 (catalog no. SAB1304232).

### Extraction of nuclear and cytoplasmic RNA

RNA was extracted from the nucleus and cytoplasm according to the Invitrogen nuclear extraction protocol and as previously described [27].

### Colony formation assay

An equal number of cells were seeded and cultured for 10 days to form colonies. Colonies were fixed with 100% ethanol, and stained with 10% Giemsa for 15 minutes and the number of colonies was determined.

### CRISPR/Cas9-mediated deletion

LAPAS1–deleted U2OS cells were generated using two distinct gRNAs. The deleted genomic region of LAPAS1 is 35,366 bp long. Two small guide RNA#1- GTTAGTTTCCTCGGCAGGTT (chr1:51,443,264) and sgRNA#2- ACATTTAGGGCCGAACCCAG (chr1:51,478,630) were planned using the CRIPSR Design Tool and cloned into pSpCas9 plasmid. The two plasmids were transfected into U2OS cells with PolyJet (Signagen). To validate the deletion, genomic DNA was isolated from the cells two weeks post transfection, with a QuickExtract kit from Epicenter, and the deletion was confirmed by sequencing (Supplementary Figure 1).

### Survival probability analysis

Kidney renal clear cell carcinoma (KIRC) Kaplan–Meier survival analysis was done by TANRIC web tool on 448 KIRC samples.

### Statistical analyses

Statistical analyses were performed using SPSS software and two-tailed Student’s *t*-test.

## SUPPLEMENTARY MATERIALS




