# Supplementary material for: A novel E2F1-regulated lncRNA, LAPAS1, is required for S phase progression and cell proliferation
Source: Oncotarget. 2021 May 25;12(11):1072–82. doi: 10.18632/oncotarget.27962 (PMC8169067; doi:10.18632/oncotarget.27962)
Supplement: Supplementary file 1 [file oncotarget-12-1072-s001.pdf]

A novel E2F1-regulated lncRNA, LAPAS1, is required for S phase progression and cell proliferation

SUPPLEMENTARY MATERIALS

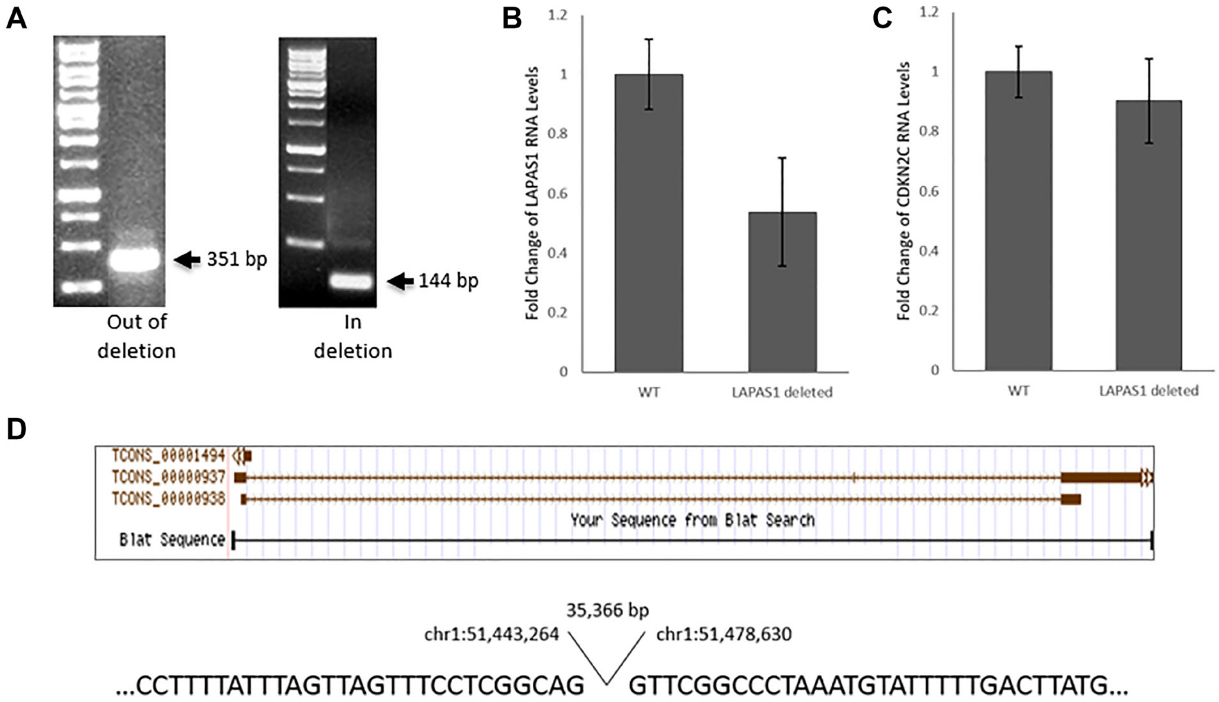

Supplementary Figure 1: Deletion of LAPAS1 generated heterozygote cells. (**A**) Genomic DNA was extracted from LAPAS1 deleted cells and the deletion of LAPAS1 was validated using PCR with primers outside the deleted region (left panel, for- GGCCCTAGTGATAGGAAGCTG, rev- TCCTTTTATCCCCCTTCAAAC) and primers within the deleted region (right panel, for- TGAACACAAAAACAGGTCCAA, rev- TCTTCTGCATTCAAAATTCCAA). Band size (using the ‘out of deletion’ primers) indicates that the deletion has occurred, while the band size using the ‘in deletion’ primers, indicates that a wt allele of LAPAS1 is retained. (**B** and **C**) RNA was extracted from WT and LAPAS1-deleted U2OS cells. Next, LAPAS1 RNA levels (B) and CDKN2C mRNA levels (C) were determined. (**D**, upper panel) Schematic representation of the LAPAS1 deletion (labeled in black). (D, lower-panel) sequencing results of LAPAS1 deletion using the PCR product described in (A, left panel) using the ‘out deletion’ primers. The exclude genomic region of LAPAS1 is 35,366 bp.

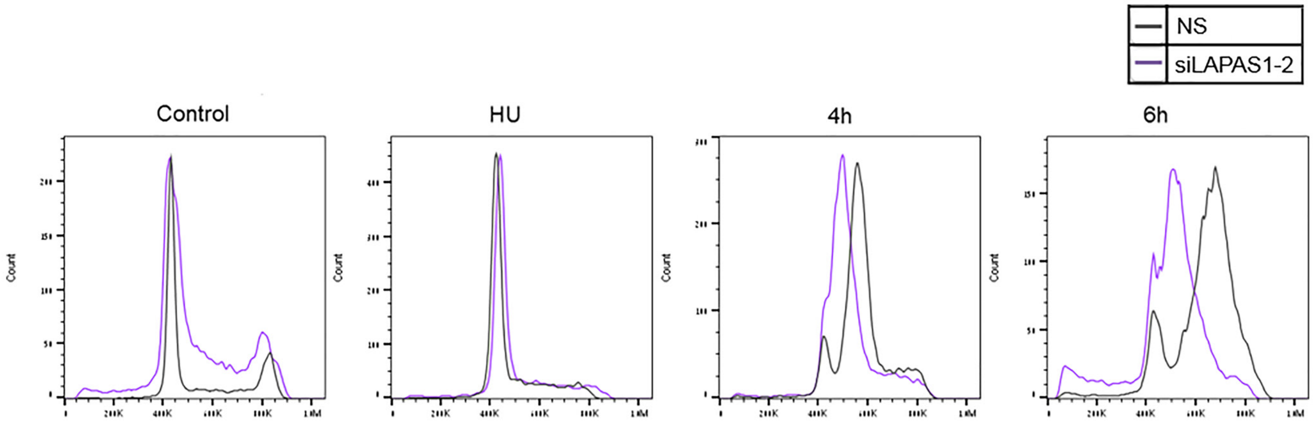

Supplementary Figure 2: An additional siRNA directed against LAPAS1 slows the progression of G1-synchronized U2OS cells through S phase. U2OS cells were transfected with either a nonspecific siRNA (NS) or siRNA directed against LAPAS1 (siLAPAS1-2). Next, cells were incubated with hydroxyurea (4 mM) for 20 hours. 48 hours post-transfection, cells were harvested or allowed to resume growth by incubation in fresh media for times indicated. Cells were analyzed by FACS using PI staining.

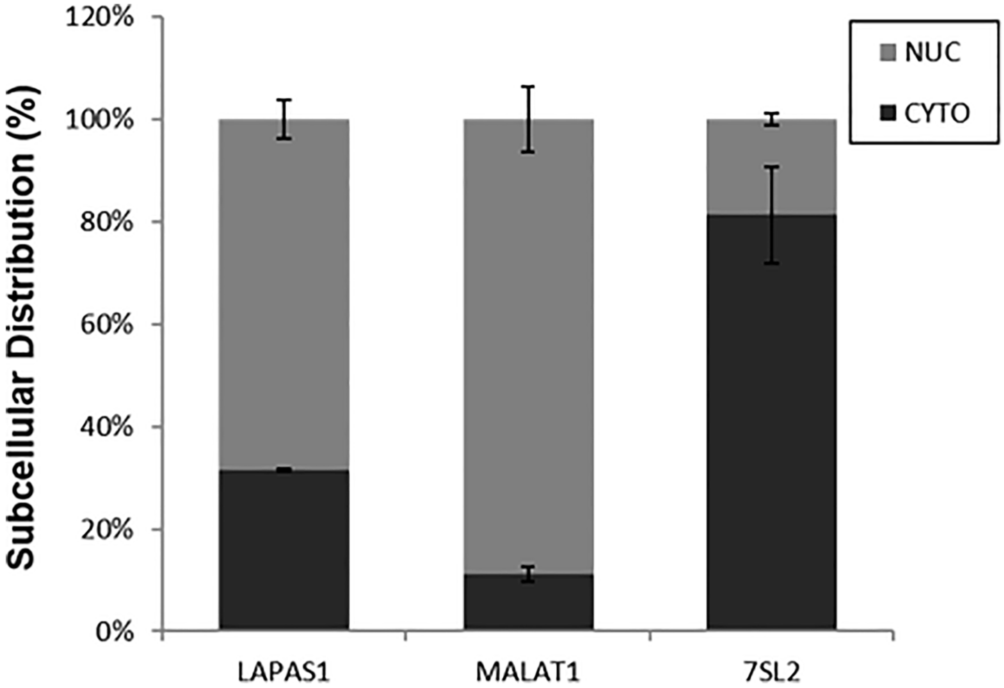

Supplementary Figure 3: LAPAS1 is localized mainly in the nucleus. RNA was extracted from nucleus and cytoplasmic fractions of U2OS cells and the levels of nuclear control transcript (MALAT1), cytoplasmic control transcript (7SL2), and LAPAS1 were determined by real-time PCR in nuclear and cytoplasmic fractions and normalized to levels of external RNA.

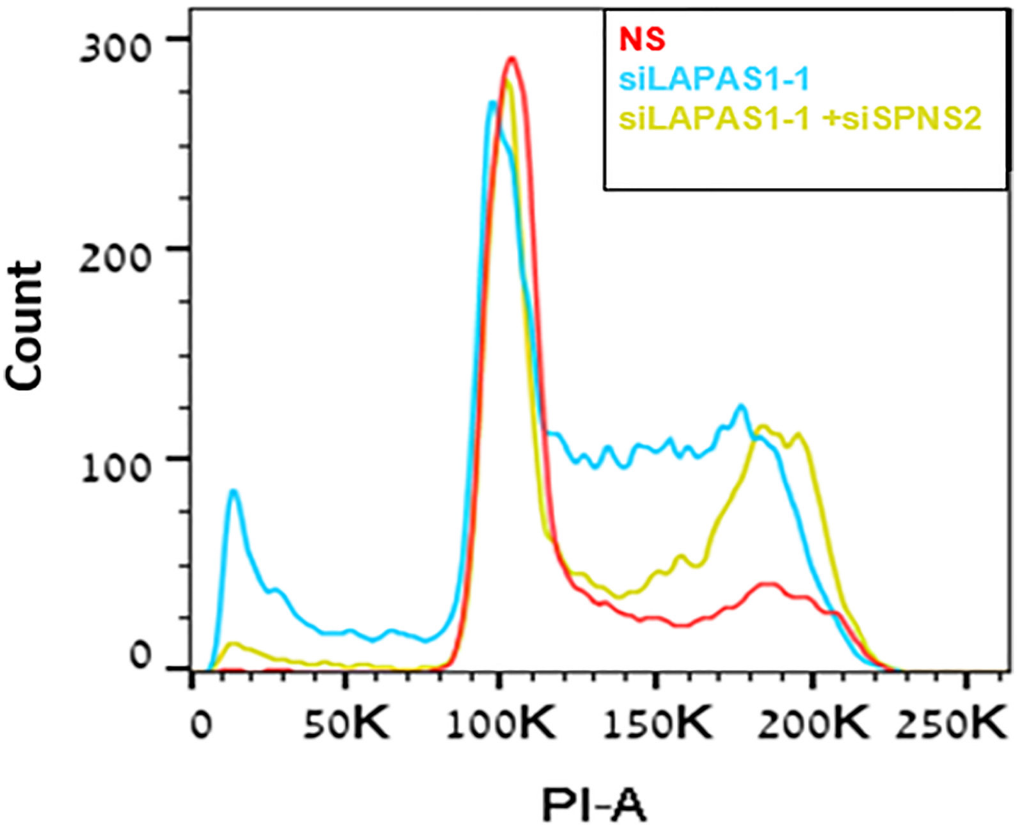

Supplementary Figure 4: Silencing of SPNS2 rescues the effect of LAPAS1 silencing on cell cycle progression. U2OS cells were transfected with either a nonspecific siRNA (NS), siRNA directed against LAPAS1 (siLAPAS1-1), or siRNA directed against LAPAS1 and SPNS2 (siLAPAS1-1 + siSPNS2). 48 hours post-transfection, cells were harvested and assayed for cell cycle distribution using FACS analysis. One representative experiment is shown.

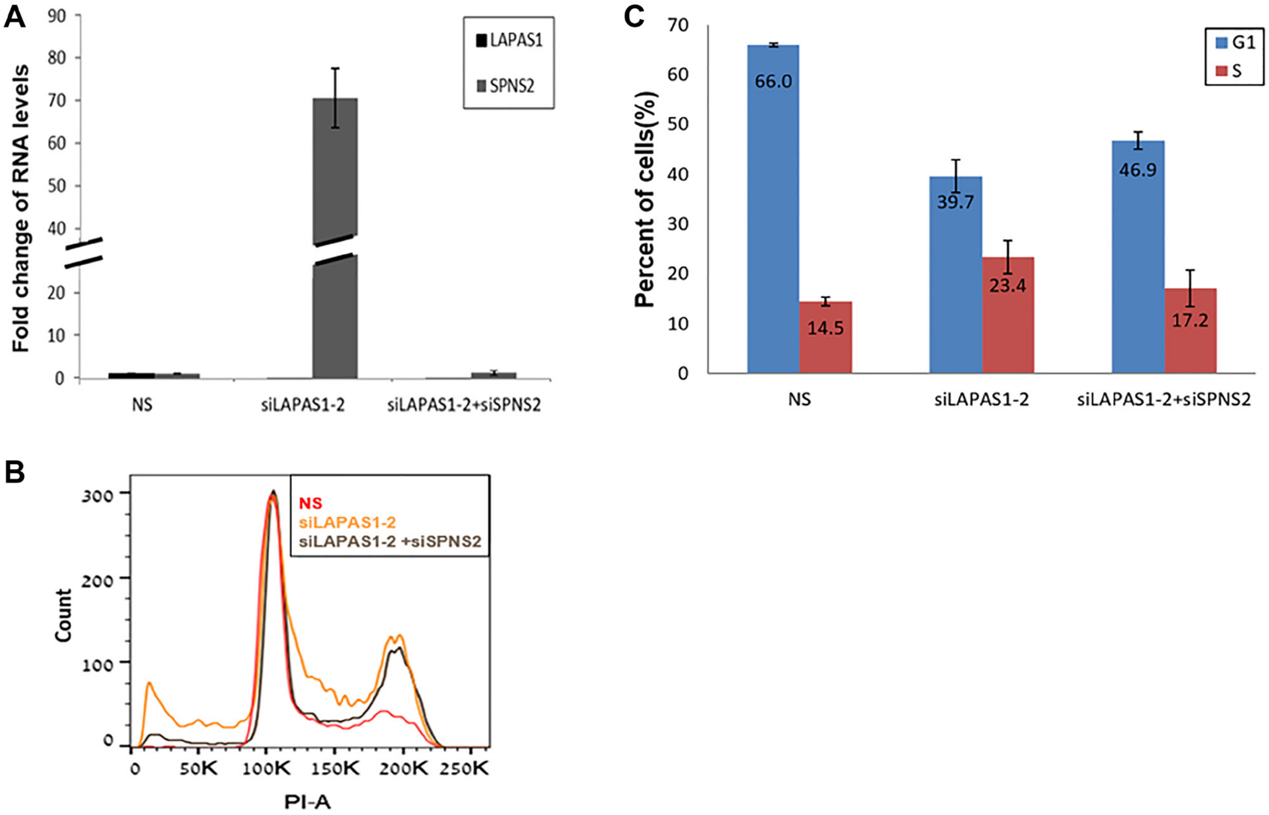

Supplementary Figure 5: Silencing of SPNS2 rescues the effect of LAPAS1 silencing, using additional siRNA, on cell cycle progression. U2OS cells were transfected with either a nonspecific siRNA (NS), siRNA directed against LAPAS1 (siLAPAS1-2) or siRNA directed against LAPAS1 and SPNS2 (siLAPAS1-2 + siSPNS2). (**A**) RNA was extracted and LAPAS1 and SPNS2 RNA levels were determined. One representative experiment is shown out of 3 repeats. (**B**) Cells were analyzed by FACS using PI staining. One representative experiment is shown. (**C**) An average of two independent FACS experiments is presented.

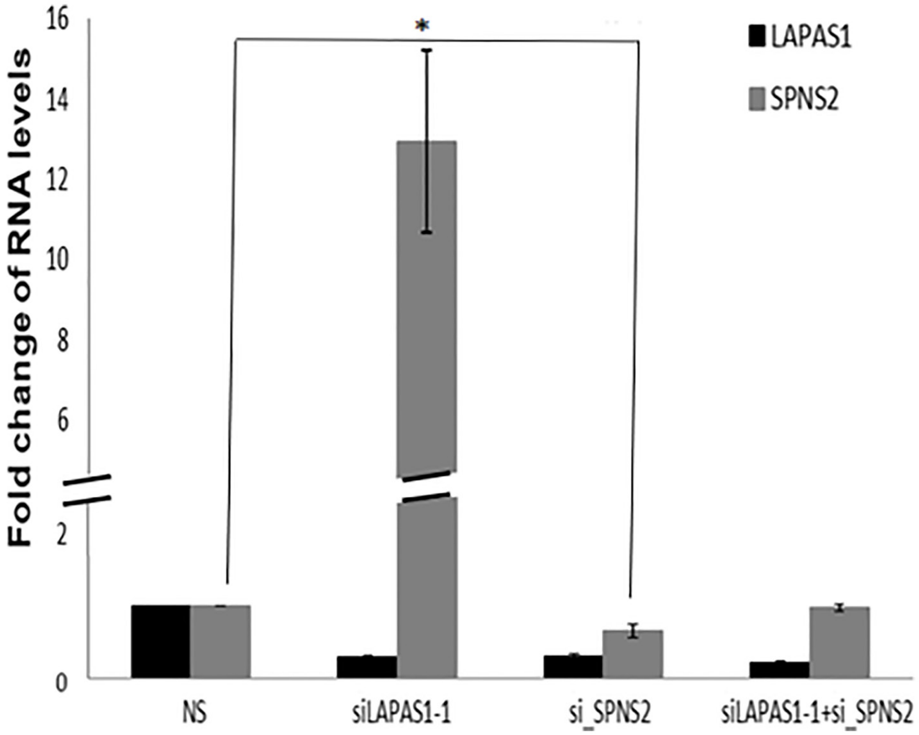

Supplementary Figure 6: RNA levels of LAPAS1 and SPNS2 following co-silencing. U2OS cells were transfected with either nonspecific siRNA (NS), siRNA targeted against LAPAS1 (siLAPAS1-1), siRNA against SPNS2, or both. Total RNA was extracted from cells on day 5 of the experiment (shown in Figure 7C), and LAPAS1 and SPNS2 RNA levels were measured using real-time PCR (qRT PCR). An average of three independent experiments is presented ($^{*}p < 0.05$, two-tailed Student's *t*-test).

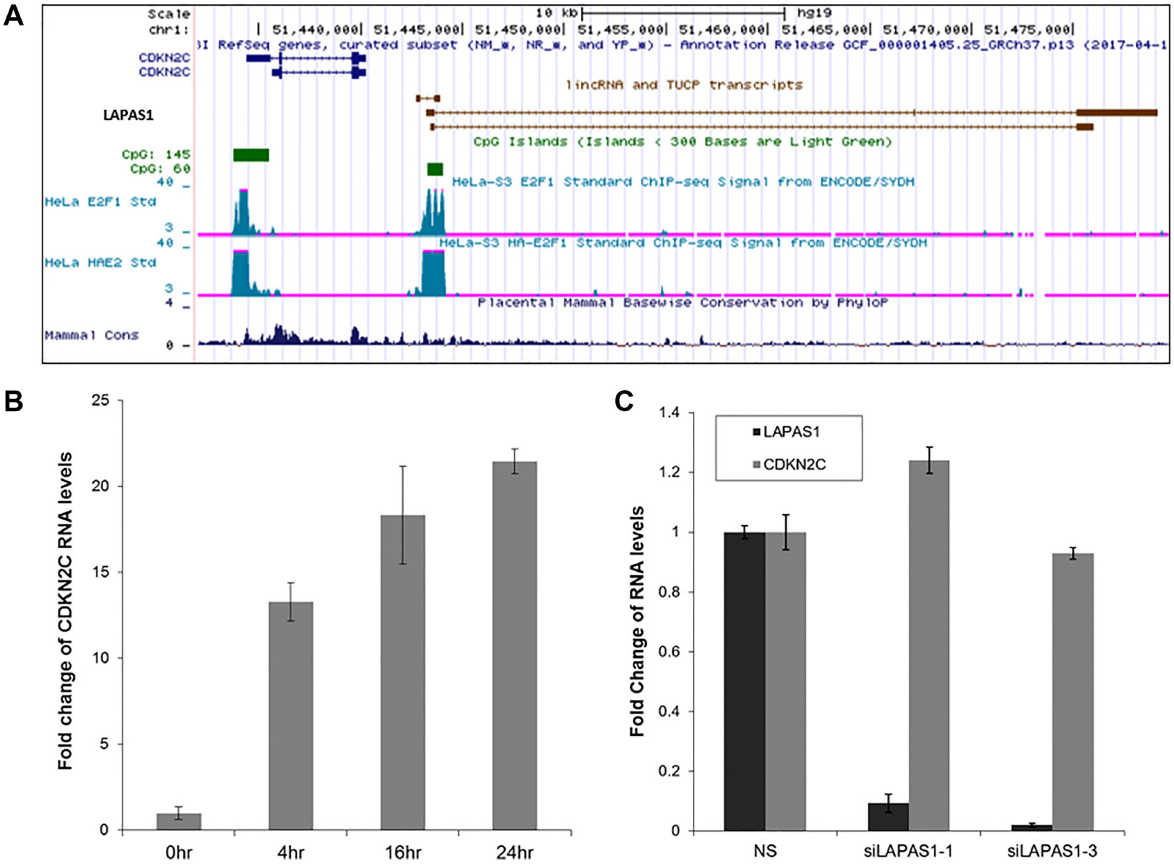

Supplementary Figure 7: CDKN2C is up-regulated by E2F and is not affected by LAPAS1 silencing. (**A**) Schematic representation of LAPAS1 on chromosome 1 is presented based on information from the UCSC Genome Browser. CpG islands are presented as green bars. E2F1 binding regions are presented as light blue peaks. (**B**) U2OS cells containing conditionally active E2F1 were induced to activate E2F1 by the addition of OHT (times indicated). RNA was extracted, and CDKN2C RNA levels were determined by real-time RT-PCR. (**C**) U2OS cells were transfected with either a nonspecific siRNA (NS) or siRNAs directed against LAPAS1 (siLAPAS1-1 or siLAPAS1-3). RNA was extracted, and LAPAS1 and CDKN2C RNA levels were determined.

Supplementary Table 1: Genes whose expression was altered upon silencing of LAPAS1. See Supplementary Table 1
